# Age-related macular degeneration and resource utilization in the Brazilian public healthcare system: a real-world retrospective study

**DOI:** 10.1186/s12886-021-02181-1

**Published:** 2021-12-13

**Authors:** Liane Touma-Falci, Carlos Augusto Moreira-Neto, Alexandre Chater Taleb, Marcela Bach Prieto, Thais Packer, Julio Cesar Barbour Oliveira, Marina Gabriela Birck, Guilherme Silva Julian, Francisco Jose Forestiero

**Affiliations:** 1 Novartis Biociências SA, Vicente Rao Avenue, 90, São Paulo, SP 04636-000 Brazil; 2 Hospital de Olhos do Paraná, Curitiba, PR Brazil; 3 Reference Centre in Ophthalmology, Universidade Federal de Goiás, Goiânia, GO Brazil; 4 IQVIA Brasil, São Paulo, SP Brazil

**Keywords:** Age-related macular degeneration, Real world data, Datasus, Antiangiogenic treatment, Public health system

## Abstract

**Background:**

Age-related macular degeneration (AMD) is a disease that causes damage in the macular region of the retina, leading to irreversible blindness. This study aims to understand the profile and care of patients with AMD and its cost at the Brazilian public health system to identify AMD-care needs.

**Methods:**

This is a retrospective observational study of AMD with real-world data from the Brazilian public healthcare system, using DATASUS claim databases. Patients with AMD were selected from 01/Jan/2014 to 31/Jan/2020; had at least one claim of ICD10 code H35.3 (Degeneration of macula and posterior pole), and were submitted to one of two procedures exclusively available for AMD patients - optical coherence tomography (OCT) and medical treatment of retinal disease (antiangiogenic); aged ≥18 years at first ICD10 claim, and presenting at least 1 year of follow-up in the database. We described patients’ characteristics, healthcare resource utilization and cost, and the antiangiogenic intravitreal treatment received by AMD patients, including the number of doses and interval time between them.

**Results:**

Patients searching for AMD treatment since 2014 were mostly females (59%), white (61%), and a mean age of 72 years. They were mainly located in the Southeast (87%), and few patients were found in the North (1%) and Central-West (1.5%) regions, probably reflecting where the Brazilian guideline to treat AMD (*Protocolo Clínico e Diretrizes Terapêuticas* - PCDT) was incorporated as routine care for AMD. The average antiangiogenic dose of 2.5 antiangiogenic therapies within a year was below the expected. Most injections had an interval time of 20 to 40 days between doses, although some patients were treated more than 100 days. Another setback is that patients traveled longer distances for OCT and antiangiogenic treatment than overall AMD-healthcare, between 10 and 100 km.

**Conclusions:**

AMD patients seem to be undertreated, as they receive a mean of 2.5 doses of antiangiogenic treatment within a year. Inequalities among regions are evident, as the Southeast and South regions comprise almost all patients receiving the treatment from the public health system, probably reflecting the region with more access to AMD care according to PCDT recommendations.

**Supplementary Information:**

The online version contains supplementary material available at 10.1186/s12886-021-02181-1.

## Background

Age-related macular degeneration (AMD) is a disease that causes damage in the macular region of the retina [1], responsible for about 8% of severe visual impairment and irreversible blindness [2]. The prevalence of AMD increases with aging, varying from 1.5% [3] to 16.7% [4] in people aged ≥50 years, 15.1% among ≥60 years old [5], and 31.5% among ≥80 years old [6] in Brazil.

Its diagnosis is based on ophthalmologic and image assessments like fundus autofluorescence, optical coherence tomography (OCT) and fluorescein angiography [7, 8]. Two main types of AMD exist: the dry AMD, that accounts for 80-90% of AMD cases; and the wet, also called neovascular AMD (nAMD), that although accounts for lower proportion of cases (10-20%), most of them (around 90%) progress with vision loss and blindness. Treatment is currently available only for nAMD [7, 9], and requires multiple visits to a healthcare center per year, which causes a burden for the patient due to the reduced quality of life, higher life stress and lower satisfaction [7], and for the health system due to economic aspects [7, 10].

Brazil is a big middle-income country in Latin America, where more than 80% of its population live in urban areas distributed throughout the whole land. As socioeconomic, political and cultural differences exist across the country, it is divided in five major regions for statistical purposes - North, Northeast, Central-West, Southeast, and South [11]. Healthcare in Brazil is mainly delivered by the Brazilian Unified Health System (Sistema Único de Saúde [SUS]), which provides access to healthcare services free of charge to the entire population, though around 75% use it exclusively.

Although several drugs are approved and have been used for nAMD treatment in Brazil since 2007, only by the end of 2018 one treatment option was incorporated for the Brazilian public health system perspective, as defined at the nAMD guideline – in Portuguese, *Protocolo Clínico e Diretrizes Terapêuticas* (PCDT). Different from clinical guidelines made by medical society, PCDT aims to standardize care and reimbursement process at public settings, based not only on clinical evidence, but also cost-effectiveness and cost-minimization analysis. Thus, PCDT has incorporated bevacizumab as nAMD treatment option at Brazilian public settings, a priori to be available for the whole country [12].

To our knowledge, no published study evaluated the current AMD care and treatment and its resource use in SUS. But understanding the profile of patients with AMD, healthcare resource utilization and cost due to the disease in the public health system contributes to identifying the AMD-care needs and improving its strategies and policies. Hence, this study aims to describe AMD patients treated at the Brazilian public health system, and to estimate the health resources utilization and cost of AMD-related care in the public health system perspective.

## Material and methods

This is a retrospective observational study of AMD with real-world data from the Brazilian public healthcare system (SUS), using DATASUS claim databases.

All data within DATASUS are anonymized and encrypted. DATASUS’ data is publicly available, and does not require approval from ethics committees, according to the Brazilian ethics Resolution n° 510/2016.

### Study population

Patients with AMD were selected from 01 January 2014 to 31 January 2020 (the last information available at data extraction). As there are no ICD-10 codes specific for AMD or its type (neovascular or dry), we considered as AMD patients those submitted to one of the two procedures in the public system exclusively available for AMD patients [13].

Thus, AMD case was defined as a patient with (a) at least one claim of ICD-10 code H35.3 (Macula and Posterior Pole Degeneration); (b) submitted to at least one of the following procedures: 02.11.06.028-3 (Optical Coherence Tomography) or 03.03.05.023-3 (Medical Treatment of Retinal Disease); (c) aged ≥18 years - to embrace cases of AMD diagnosed before the age of 60; and (d) presenting at least 1 year of data in the database - to make sure that the patient is SUS-exclusively dependent and does not seek SUS only for high-cost medication. Exclusion criteria were (a) oncologic patients (ICD-10 codes or under chemotherapy or radiotherapy procedures), or (b) inconsistent or excessive missing data.

### Data source

Procedures performed within the SUS structure are recorded in DATASUS, by the Brazilian Ministry of Health Department of Informatics. The reimbursement administrative database contains information about inpatient and outpatient healthcare.

For this study, we used both SIH (*Sistema de Informações Hospitalares* [Inpatient Information System]) and SIA (*Sistema de Informações Ambulatoriais* [Outpatient Information System]) databases. SIH and SIA are not linked by a unique patient identifier, thus we used a probabilistic record linkage to achieve longitudinal patient data. The linkage considers date of birth, ZIP code, ICD-10 historical data, and other demographic information in databases, which are described elsewhere [14]. Patients entered only once in this study, when the first claim of ICD-10 code H35.3 appeared after January 2014.

Besides, we used CNES (*Cadastro Nacional de Estabelecimentos de Saúde* [National Register of Health Establishments]) database to describe institutions.

### Other variables

For this study, we considered the following definitions:

Age was defined as the age at the first claim of ICD-10 code H35.3. Follow-up is the time from the first claim of H35.3 after 01 January 2014 up to the last patient information (last claim) available at the database, stratified by those who received medical treatment for retinal disease and those who did not.

AMD-related hospitalization was based on ICD-10 code H35.3, while outpatient visits related to AMD were based on the same ICD-10 code H35.3, or any ICD-10 from H00-H59 (eye-related ICDs), or Z01.0 (Encounter for examination of eyes and vision), or AMD specific procedures – 02.11.06.028-3 (Optical Coherence Tomography) and 03.03.05.023-3 (Medical Treatment of Retinal Disease). Antiangiogenic therapy was defined as the procedure 03.03.05.023-3 - Medical treatment of retinal disease. Interval between doses of antiangiogenic therapy was defined as the time (days) between one claim of the procedure to the subsequent one.

Distance was calculated as the Euclidean distance (km) from two zip codes: patient’s residence and the healthcare facility or tomography or antiangiogenic treatment institution, as applicable.

### Data analysis

This is a population-based study that covers all the population using SUS, so no sample size was calculated. This is study is descriptive in nature, so no hypothesis test was performed. Categorical variables are expressed as absolute number and proportion, and continuous as measures of central tendency (mean and/or median) and data distribution (standard deviation [SD] and/or interquartile range [IQR]). We used Python version 3.7.7 (Python Software Foundation) for data analysis. No imputation methods were used for missing data.

## Results

From 01 January 2014 to 31 January 2020 (6 years of study period), 29,808 patients with AMD seeking SUS for healthcare were identified. We excluded 6777 (33%) due to the following criteria: history of cancer (1066), less than 1 year of follow-up (3468), or with inconsistence data (2243). All 23,031 AMD patients included in this study had ambulatorial (outpatient) visit due to the disease, and 62 (0.3%) were also hospitalized (inpatient) with ICD-10 H35.3 as primary or secondary cause of admission.

### Profile of AMD patients

The profile of AMD patients treated at the Brazilian public health system from January 2014 to January 2020 are shown in Table 1. Rate of AMD patients was higher in the Southeast region, which represent the vast majority of the study population (87%); whereas very few cases were found in the other regions. Most patients were older than 60 years, and none were younger than 50. Similar demographic characteristics were found around all Brazilian regions: most patients were female and White, with a mean age of 72 years. Overall, about half of the AMD patients were followed up by approximately 4-6 years during the study period, although the North region had the highest proportion (18.6%) of patients with less than 2 years of follow-up.

**Table 1:** Demographic characteristics of AMD-patients treated at the Brazilian public health system since 2014, and the follow-up period of treatment

	Brazil	North	Northeast	Central-West	Southeast	South
**AMD-patients**, N (%)	23,031	261 (1.1)	628 (2.7)	354 (1.5)	20,049 (87.1)	1739 (7.6)
**AMD patients per 100,000 older adults**^a^	79	16	9	19	146	36
**Age** (years), mean (SD)	72.45 (7.39)	70.38 (7.38)	71.28 (7.19)	72.10 (7.70)	72.66 (7.37)	72.20 (7.46)
**Age strata**, N (%)						
≤ 50 years-old	0 (0.0)	0 (0.0)	0 (0.0)	0 (0.0)	0 (0.0)	0 (0.0)
51- 60 years-old	27 (0.1)	10 (2.1)	4 (0.3)	1 (0.2)	11 (0.1)	1 (0.01)
61-70 years-old	10,329 (44.9)	273 (56.5)	775 (50.2)	266 (47.4)	7573 (43.5)	1442 (47.5)
71-80 years-old	9091 (39.5)	143 (10.8)	588 (38.1)	208 (37.1)	7019 (40.3)	1133 (37.3)
81-90 years-old	3285 (14.3)	52 (10.8)	162 (10.5)	80 (14.3)	2566 (14.7)	425 (14.0)
≥ 91 years-old	299 (1.3)	5 (1.0)	16 (1.0)	6 (1.1)	236 (1.4)	36 (1.2)
**Female,** N (%)	13,615 (59.1)	141 (54.0)	370 (58.9)	196 (55.4)	11,846 (59.1)	1062 (61.1)
**Race,** N (%)						
White	14,182 (61.6)	161 (61.7)	401 (63.9)	206 (58.2)	1233 (61.5)	1081 (62.2)
Mixed	3300 (14.3)	36 (13.8)	91 (14.5)	43 (12.1)	2864 (14.3)	266 (15.3)
Black	5549 (24.1)	64 (24.5)	136 (21.7)	105 (29.7)	4852 (24.2)	392 (22.5)
**Follow-up of treated patients**^b^ (*N* = 1460)						
**Follow-up**^b^ (years), mean (SD)	0.73 (0.88)			0.57 (0.50)	0.79 (0.97)	0.57 (0.34)
**Follow-up strata**^b^, N (%)						
< 6 months	660 (45.2)			25 (49.0)	533 (46.9)	102 (37.5)
6-12 months	616 (42.2)			24 (47.1)	426 (37.5)	166 (61.0)
1-2 years	77 (5.3)			–	74 (6.5)	–
> 2 years	43 (2.9)			2 (3.9)	40 (3.5)	1 (0.4)
> 3 years	64 (4.4)			–	64 (5.6)	–
**Follow-up of non-treated patients**^b^ (*N* = 21,571)						
**Follow-up**^b^ (years), mean (SD)	0.36 (0.66)	0.25 (0.30)	0.25 (0.57)	0.16 (0.20)	0.41 (0.72)	0.26 (0.30)
**Follow-up strata**^b^, N (%)						
< 6 months	16,177 (75.0)	471 (92.4)	1379 (89.3)	368 (76.2)	11,716 (72.0)	2243 (81.1)
6-12 months	4406 (20.4)	38 (7.5)	132 (8.5)	111 (23.0)	3610 (22.2)	515 (18.6)
1-2 years	371 (1.7)	1 (0.2)	3 (0.2)	3 (0.6)	363 (2.2)	1 (0.0)
2-3 years	231 (1.1)	–	4 (0.3)	1 (0.2)	224 (1.4)	2 (0.1)
> 3 years	386 (1.8)	–	27 (1.7)	–	355 (2.2)	4 (0.1)

^a^Number of AMD patient aged 60 years or more, per 100,000 inhabitants aged 60 years or more, according to IBGE estimates in 2019; ^b^Follow-up since the first claim of AMD ICD10 code (H35.3)

### Healthcare resource utilization and costs of AMD-related care

**Fig. 1:**
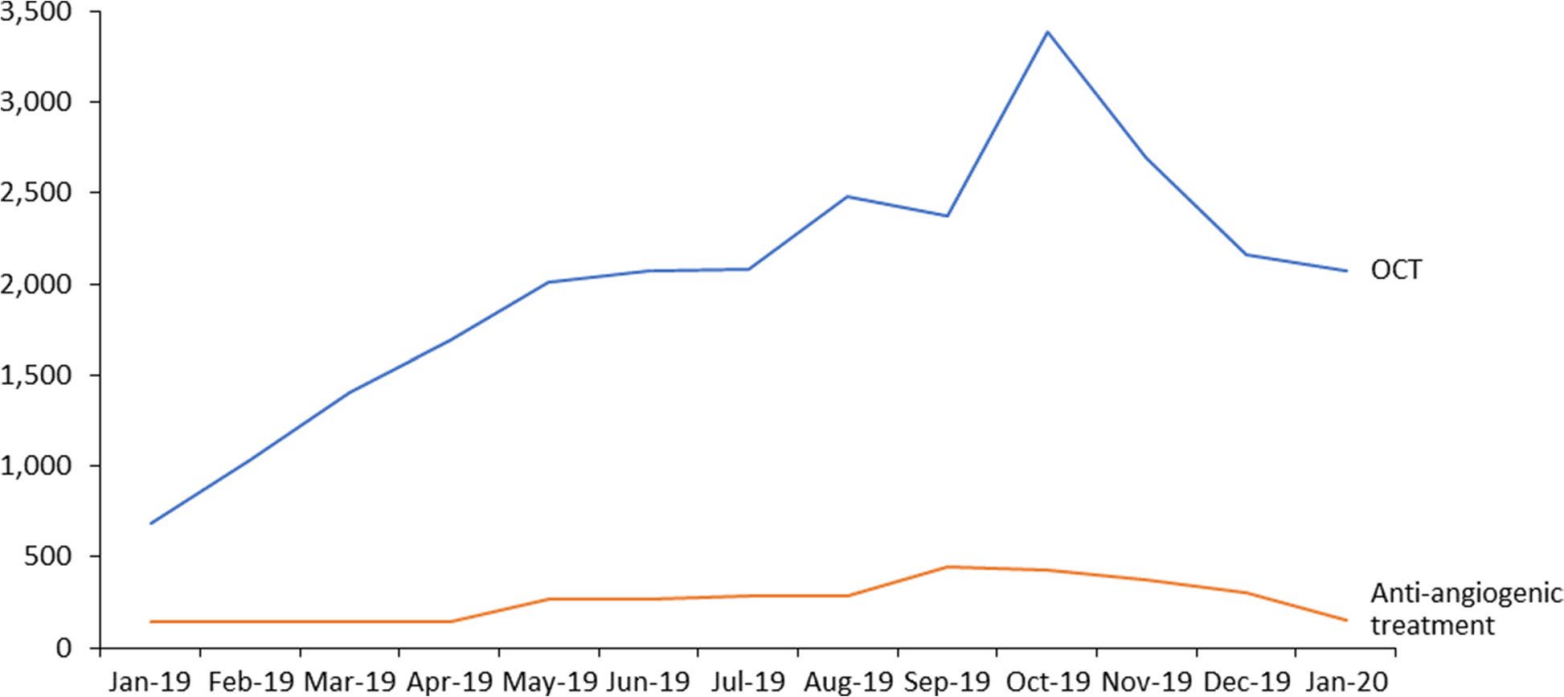
Number of OCT and treatment procedures throughout time after PCDT

During the 6 years of the study period, 26,155 OCT were performed by all AMD-patients included. After PCDT approval in November 2018, only 1460 AMD-patients performed the antiangiogenic injection, accounting for 3372 procedures. Other eye-related procedures are also described in Table 2. The number of OCT and retinal treatment (antiangiogenic intravitreous injection) showed a growing pattern since they were incorporated in SUS, although it is more outstanding for OCT than for retinal treatment (Fig. 1).

**Table 2:** Eye-related procedures performed by AMD-patients treated at the Brazilian public health system since 2014 (*N* = 23,031)

Eye-related procedures (SIGTAP code)	N
Optimal coherence tomography (02.11.06.028-3)	26,155
Medical treatment of retinal disease (03.03.05.023-3)	3372
Tonometry (02.11.06.025-9)	161,836
Mapping of retina (02.11.06.012-7)	136,678
Colorful binocular retinography (02.11.06.017-8)	33,000
Fluorescent binocular retinography (02.11.06.018-6)	29,016
Fundoscopy (02.11.06.010-0)	25,190
Intravitreous injection (04.05.03.005-3)	24,339
Laser photocoagulation (04.05.03.004-5)	21,868
Ultrasonography of the ocular globe / orbit (monocular) (02.05.02.008-9)	14,092

The 23,031 AMD-patients accounted for 938,780 outpatient procedures, with a median annual number of outpatient procedures per patient of 4.3 (IQR 1.8-9.3). Regarding specifically to AMD-procedures, the annual median number of OCT and antiangiogenic injection per patient were 1.25 (0.93-1.64) and 2.37 (1.35-3.43), respectively. Among the AMD patients in the inpatient dataset, the median number of hospitalization was only one (IQR 1-2) during the study period, with a median length of stay per hospitalization of 1 day (Table 3), in which the most common procedure performed was vitrectomy.

**Table 3:** Healthcare resource utilization by AMD patients treated at the Brazilian public health system since 2014

	Mean (SD)	Median (IQR)
**Outpatient (N = 23,031)**		
Number of procedures per patient/year	13.52 (32.69)	4.33 (1.83-9.31)
Number of OCT per patient/year	1.36 (0.55)	1.25 (0.93-1.64)
Number of antiangiogenic therapies per patient/year	2.50 (1.36)	2.37 (1.35-3.43)
**Inpatient (*N*** **= 62)**		
Number of hospitalizations per patient - total	1.42 (0.66)	1.00 (1.00-2.00)
Number of hospitalizations per patient/year	0.87 (0.88)	0.56 (0.29 – 0.94)
Length of stay per hospitalization, in days	1.11 (0.32)	1.00 (1-1)

The median annual costs (in Brazilian currency, BRL) for AMD-related outpatient visit were R$ 687.00 per patient (IQR 351.58-1155.83), whereas only the procedures performed during those visits accounted for a median annual cost of R$ 219.13 (IQR 84.23-514.89). For AMD specific procedures, median annual cost per patient of OCT and antiangiogenic treatment were BRL 51.53 (IQR 44.58-81.49) and R$ 186.19 (IQR 105.00-277.05), respectively. Among the 62 AMD-patients hospitalized with ICD-10 H35.3 as primary or secondary case of admission, the median annual cost of hospitalization due to AMD was R$ 2624 per patient (IQR 1703.75-3420.25) with a median annual cost of only procedures performed of R$ 1203.88 per patient (IQR 586.73-2657.57), mainly reflecting the cost of vitrectomy procedures (Table 4).

**Table 4:** Cost (BRL, R$) of AMD-related outpatient and inpatient visits at the Brazilian public health system, since 2014

	Mean (SD)	Median (IQR)
**Outpatient (*n*** **= 23,031)**		
Cost of outpatient visit per patient/year	809.07 (587.95)	687.77 (351.58-1155.83)
Cost of overall outpatient procedures per patient/year	412.48 (558.78)	219.13 (84.23-514.89)
Cost of OCT per patient/year	67.27 (31.85)	51.53 (44.58-81.49)
Cost of antiangiogenic per patient/year	201.81 (109.89)	186.19 (105.00-277.05)
**Inpatient (*n*** **= 62)**		
Cost of hospitalization per patient/year	2988.18 (1828.33)	2624 (1703.75-3420.25)
Cost per overall inpatient procedures per patient/year	1943.80 (2047.73)	1203.88 (586.73-2657.57)
Cost per day of hospitalization	2903.72 (1875.81)	2624 (1703.00-3142.75)

### Institutions of AMD-healthcare and spatial analysis

**Fig. 2:**
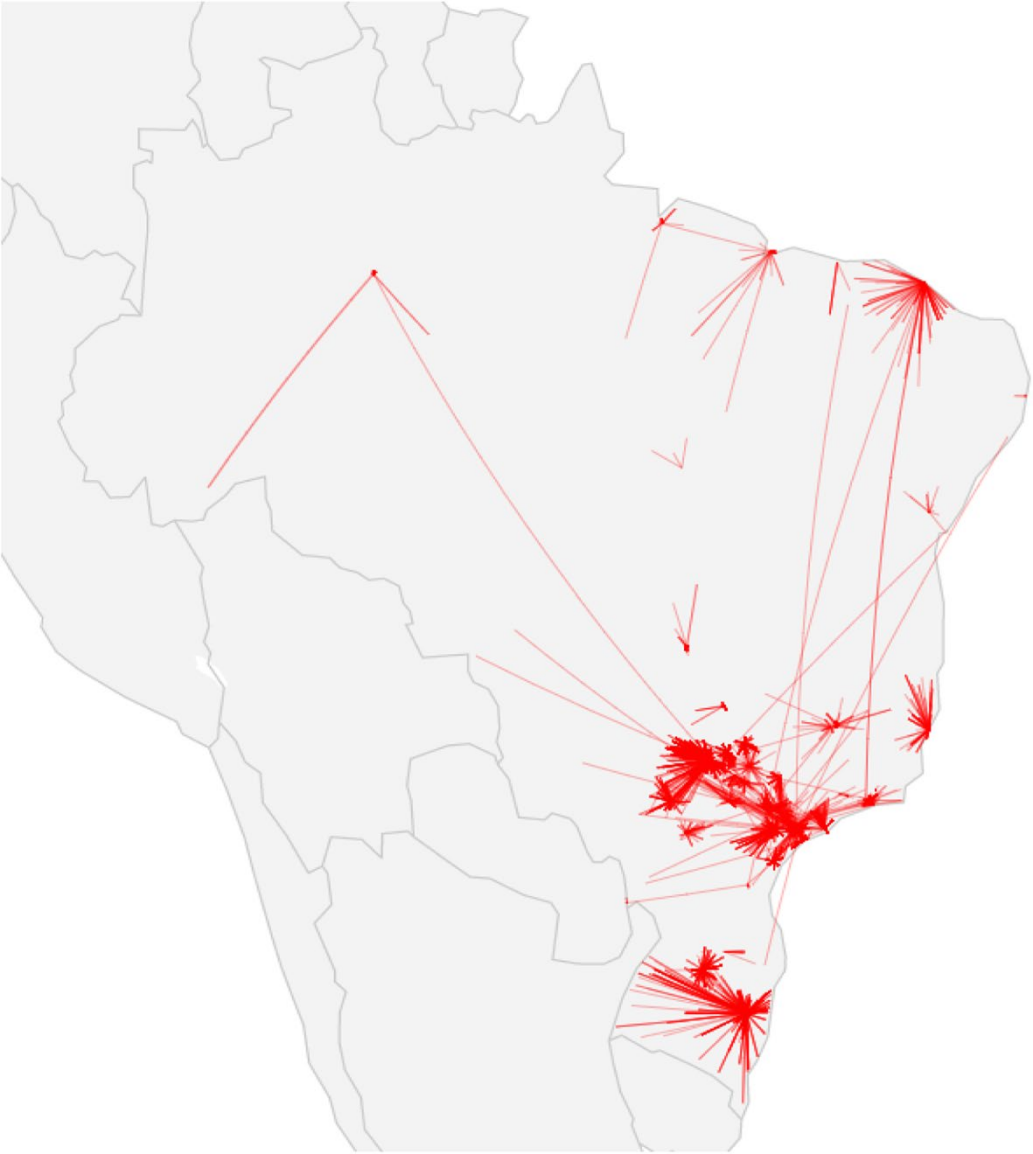
Route and distance travelled from patient’s residence to AMD healthcare facility

**Fig. 3:**
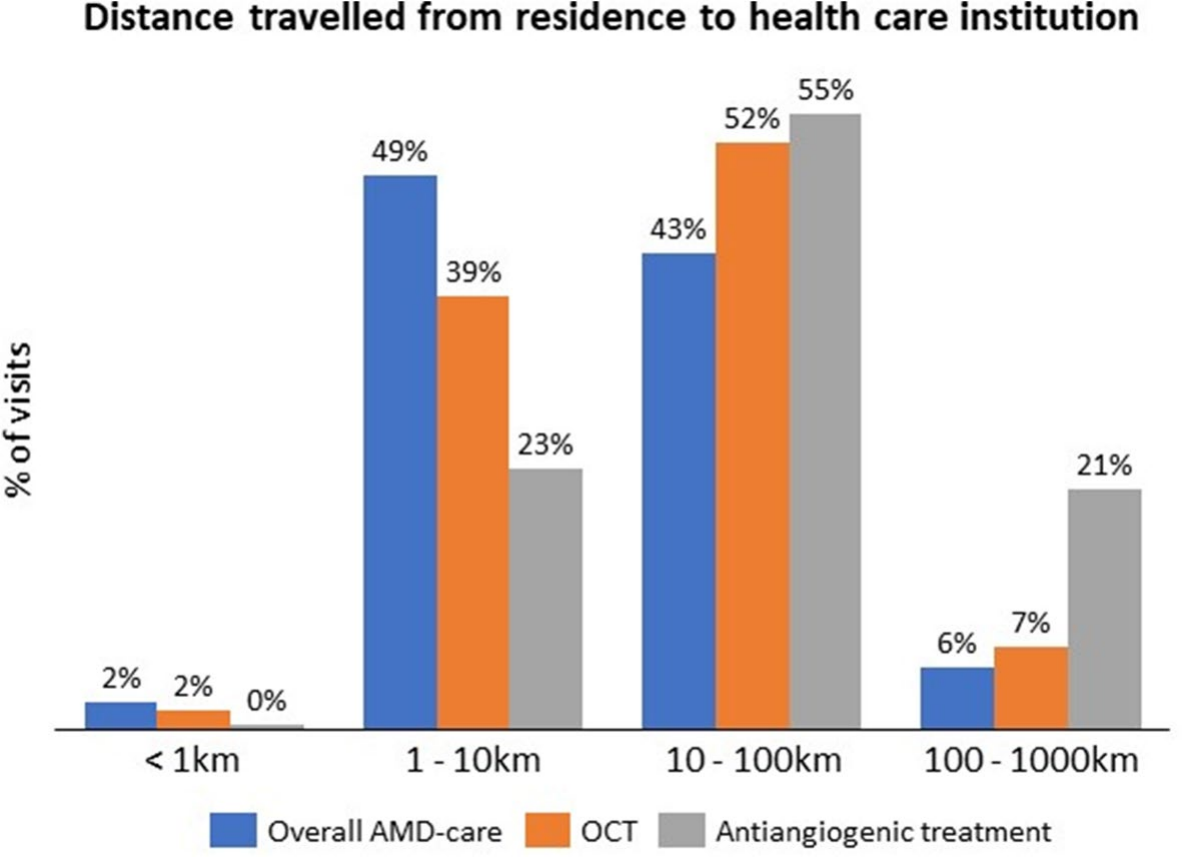
Distribution of the distances travelled by AMD patients from their residences to the institutions performing any AMD-healthcare (blue), OCT (orange) and to antiangiogenic treatment institution (grey)

A traveling map displayed the routes from patient’s residence to Brazilian healthcare institution where they have sought AMD care which also shows that most institutions and AMD-related care are gathered in the South and Southeast regions (Fig. 2). Overall, patients traveled from 1 to 100 km to access healthcare for AMD. Longer distances were traveled for OCT and antiangiogenic treatment than for overall AMD-healthcare, as they usually performed these procedures more than 10 km from their residence. This is worst for antiangiogenic treatment, as more than 20% of the injections were done 100-1000 km away (Fig. 3). Mean distance traveled for any AMD-healthcare was 28.4 (SD 76 km), compared to 34.3 (SD 68) km for OCT procedure and 68.9 (SD 75) km for antiangiogenic treatment. The North region showed lower mean distance compared to the South (14.7, SD 88 vs 53.8, SD 89 km for any AMD-healthcare; and 20.6, SD 138 vs 59.8, SD 88 for OCT procedure) (Additional Table 1).

The distribution and description of institutions that performed at least one OCT during the study period among the AMD patients included in this cohort is shown in Additional Table 2. Most institutions were general hospitals and clinics, and about half of them are regional administrative hospitals, followed by state hospitals.

### Description of antiangiogenic treatment

Most AMD-patients receiving an intravitreous injection according to PCDT for AMD care were from the Southeast county region, whereas no patient performed it at the North. Most of these patients (59%) received one or two injections during the 1 year of follow-up period after PCDT approvals, with a mean interval between sequential doses of 52 (SD 50) days. Central-West region showed the highest mean interval between doses (69 ± 63 days), whereas the South showed the lowest interval (41, SD45 days) (Table 5). Similar mean interval time was found among doses, although the interval between the 3rd to the 4th and the 7th to the 8th antiangiogenic doses showed more distinct times (65 SD 64 and 23 SD 13 days, respectively). Overall, AMD-patients were receiving antiangiogenic injection according to the PCDT recommendation from 20 to 40 days between one dose to the following one, although some patients were treated more than 100 days between doses (Table 6).

**Table 5:** Description of antiangiogenic treatment, according to PCDT recommendation, of AMD-patients in the Brazilian public health system

	Brazil	North	Northeast	Central-West	Southeast	South
**AMD patients with at least one antiangiogenic therapy,** N (%)	1460	0	4 (0.2)	55 (3.7)	1129 (77.3)	272 (18.6)
**Doses of antiangiogenic therapy per patient,** median (IQR)	2 (1-3)	–	2 (1-4)	2 (1-3)	2 (1-3)	3 (1-4)
**Doses strata**, N (%)						
≤ 2 doses	866 (59.3)	–	2 (50.0)	36 (65.5)	698 (61.8)	130 (47.8)
3 – 4 doses	496 (34.0)	–	1 (25.0)	17 (30.9)	380 (33.6)	98 (36.0)
5 – 6 doses	76 (5.2)	–	0 (0.0)	2 (3.6)	41 (3.6)	33 (12.1)
7 – 8 doses	17 (1.2)	–	1 (25.0)	0 (0.0)	8 (0.7)	8 (2.9)
≥ 9 doses	5 (0.3)	–	0 (0.0)	0 (0.0)	2 (0.02)	3 (1.1)
**Interval-time between doses of antiangiogenic therapy (days),** median (IQR)	31 (28-61)	–	45.5 (30.75-66.5)	39 (34.25-69.5)	35 (30-61)	28 (19-39)
**Interval-time strata**, N (%)						
< 20 days	174 (12.7)	–	0 (0.0)	3 (6.0)	37 (4.6)	134 (26.3)
20 – 40 days	704 (51.5)	–	2 (50.0)	25 (50.0)	427 (53.1)	250 (49.1)
40 – 60 days	134 (9.8)	–	1 (25.0)	4 (8.0)	72 (9.0)	57 (11.2)
60 – 80 days	143 (10.4)	–	0 (0.0)	6 (12.0)	117 (14.6)	20 (3.9)
80 – 100 days	50 (3.6)	–	1 (25.0)	2 (4.0)	39 (4.9)	8 (1.6)
> 100 days	161 (11.7)	–	0 (0.0)	10 (20.0)	111 (13.8)	40 (7.9)

**Table 6:** Detailed description of interval time between each dose of antiangiogenic injection

	1st to 2nd	2nd to 3rd	3rd to 4th	4th to 5th	5th to 6th	6th to 7th	7th to 8th
**Interval-time between doses of antiangiogenic therapy (days),** median (IQR)	34 (28-61)	33 (28-53)	35 (21-90.5)	28 (14-52)	28 (14-61)	30 (20-64)	21.5 (14-28.75)
**Interval-time strata**, N (%)							
< 20 days	47 (7.5)	37 (8.7)	35 (22.2)	27 (35)	17 (45.9)	5 (25.0)	5 (41.7)
20 – 40 days	354 (57.1)	239 (56.3)	49 (31.0)	26 (33.8)	16 (43.2)	8 (40.0)	6 (50.0)
40 – 60 days	61 (9.8)	56 (13.2)	9 (5.7)	6 (7.8)	0 (0.0)	1 (5.0)	1 (8.4)
60 – 80 days	70 (11.2)	34 (8.0)	19 (12.0)	10 (13.0)	6 (16.2)	4 (20.0)	0 (0.0)
80 – 100 days	16 (2.5)	14 (3.3)	14 (8.9)	2 (2.6)	3 (8.1)	0 (0.0)	0 (0.0)
> 100 days	72 (11.6)	44 (10.3)	32 (20.2)	6 (7.8)	5 (13.5)	2 (10.0)	0 (0.0)

## Discussion

Brazil has a universal and public healthcare system – SUS, which recently standardized nAMD care. Since its incorporation, nAMD treatment should be available for the entire population across the country regions. Though we could not classify as neovascular or dry AMD, as only the first has treatment available, most patient included in this study could be nAMD patients, especially those on anti-VEGF treatment. Patients searching for AMD care at Brazilian public institutions since 2014 were mostly females (59%), white (61%), and aged about 72 years. They were mainly located in the Southeast, and few patients were found in the North (1%) and Central-West (1.5%) regions, probably reflecting where the PCDT could be properly incorporated as routine care for AMD. The average dose was below expected, as patients received 2.5 antiangiogenic therapies within a year, with an interval time of 20 to 40 days between doses, although some patients were treated more than 100 days between doses. Moreover, patients traveled long distances for OCT and particularly for antiangiogenic treatment, longer than overall AMD-healthcare.

Most of the Brazilian population was treated for AMD after 60 years of age in the public system, which might be explained by the restriction imposed for the use of OCT and antiangiogenic therapy, only approved for older adults [10]. Most studies ﻿showed the pooled prevalence of AMD, mapped to an age range of 45–85 years, based on a meta-analysis [15] and primary care for people older than 50 years ﻿can lead to good treatment outcomes by detecting early signs of AMD and the necessity of prompt referral to an ophthalmologist [9]. Hence, the age restriction of OCT and antiangiogenic procedures probably leaves to many undiagnosed cases of AMD in younger adults.

A previous multiethnic population-based study in the USA, the prevalence of early AMD was highest in European ancestry people, compared with Hispanics, Asians (Chinese), and African Americans [15]. Ethnic origin is not likely to be a factor in Brazil due to the widespread mixture of races. However, white people composed around 60% of AMD-patients studied here and there was a higher frequency of AMD in females. AMD was similarly more prevalent in females in two centers in Pernambuco, Brazil [16]. Additionally, female and genetic backgrounds were independently associated with early AMD incidence over a 15 years study in Australia [17]. Another study ﻿found no gender effect for AMD prevalence when all races were considered globally [15]. The reasons for this scenario are still unclear. Interestingly, ﻿one study observed [4] that females had higher odds of presenting visual impairment than males, leaving some evidence that females are more susceptible to AMD. However, other reasons for gender differences cannot be discarded, such as females’ better health care consciousness compared to males.

As stated before, AMD treatment was incorporated in SUS by the Brazilian PCDT at the end of 2018. It included solely bevacizumab as drug option, with the following administration scheme: the fixed monthly model, in which a patient would receive 12 injections per year; the pro re nata model (PRN, “as needed”), where a patient is monitored monthly and only receives injections if needed, with or without three loading doses; and the treat and extend model, where patients are submitted to monthly injections until physicians deemed unnecessary, and spacing doses varies from four to twelve weeks [18]. Our findings showed that few people diagnosed at SUS started antiangiogenic treatment according to PCDT recommendation, which could be explained by contraindication of bevacizumab use for many patients, or unavailability of the drug in some regions where PCDT could not be properly implemented. Patients not treated by SUS standard of care could be either untreated, or treated with other drugs, accessed by administrative process or injunctions. This highlights that even though the PCDT was approved to improve treatment accessibility throughout the country, some patients may be still facing barriers to access AMD treatment.

Moreover, most patients treated at SUS received 2.5 doses of antiangiogenic treatment within a year, suggesting an incomplete therapy for the Brazilian population under PCDT care, as most of them should be taking monthly injections at least at the beginning of AMD treatment or monthly as suggested by another study [10]. Reasons are unclear, but it could be related to early discontinuation due to adverse events or ineffectiveness of the drug. Another hypothesis is the hurdle to access healthcare, as specialized institutions are mostly located in some few cities, so patients need to travel long distances, leading to partial or total treatment abandonment. Also, considering the overload of public ophthalmologic centers, physicians might be preferring the “PRN model” or “treat and extend model”, which could partially explain the lower rates of injections, although it would still be expected at least three loading dose per patient.

Regional differences were found in this study. Though it is a federal guideline, the availability of specialized institutions that incorporated PCDT recommendation seems concentrated in some few places, and mostly in Southeast and South region, imposing a barrier to access AMD treatment. Besides, one-fourth of the Southern region patients received two doses of antiangiogenic therapy in less than 20 days, while the average for the other regions was between 20 and 40 days. The presence of bilateral AMD in these patients or a higher percentage of general hospitals performing the treatment in the South region could explain the results. Indeed, the South region was only behind the Southeast region in number of hospitals offering antiangiogenic therapy. Alternatively, the distances were shorter in the South for institutions performing antiangiogenic treatment, as observed here. The North region, on the other hand, was not performing any antiangiogenic therapy, probably due to an absence of specialized centers performing the treatment according to the PCDT recommendation.

Because the inclusion of OCT in SUS was only in 2019, the high number of tonometry and mapping of the retina treatments performed during the study period could reflect the exams used for the diagnosis and disease monitoring of AMD prior to PCDT. However, the average of OCT performed after its introduction was below the recommended [12], as it should be performed before the antiangiogenic injection.

The current guideline recommends vial sharing for the AMD treatment, which was found to represent a cost saving for the system, and consequently increasing the availability of the drug for patients treated at the public system [10, 19, 20]. We found a low annual cost of antiangiogenic treatment per patient, that mainly reflects the low number of injections performed per patient, different from the 12 injections per patient estimated in previous study [10]. Worth noting that previous studies showed that the process of vial sharing and repacking doses in syringes, its transportation and storage, are related to lowering the quality of the product, which increases the odds of side effects [21, 22]. Adverse drug reactions are likely to increase the annual costs of AMD care, besides leaving to worst prognosis and drop out.

There were few cases of in-hospital care, but vitrectomy was the most common procedure performed. It was not possible to evaluate the reason for its recommendation, but it could be explained as the single option for AMD treatment or slowing disease progression, or as an adjuvant therapy in anti-VEGF treatment [23–25].

Although this study could not evaluate the trends in patient’s access to AMD care in the Brazilian public system, it does indicate that AMD patients treated by these health services receive suboptimal treatment, much less than ideal, with the currently available treatment. Nonetheless, as PCDT recommendations were not equally implemented in the different county, optimizing the diagnosis and treatment of AMD in each region is still a major challenge.

### Strengths and limitations

This study relies on real world data from all patients using SUS for healthcare, which accounts for around 75% of Brazilian people. Also means that it includes a diverse population regarding race and country regions.

On the other hand, this study has limitations. The study is retrospective and based on reimbursement data, relying on the quality and the correct filling of non-mandatory data. Although we used a proxy strategy for the ICD-10 code and the available procedures only for AMD patients, misclassification of AMD is possible, if OCT and/or antiangiogenic treatment was performed for another eye condition, such as macular hole and diabetic macular edema. Besides, in-hospital care was only assessed if ICD-10 code H35.3; hence, hospitalization related to AMD but without the ICD-10 code as primary or secondary cause of admission was not included in the analysis. Also, due to the data’s nature, we could not assess unilateral or bilateral AMD and the disease staging.

Worth noting that this data does not reflect AMD’s prevalence around Brazilian regions, but the distribution of patients who had access to AMD care according to the PCDT recommendation. AMD patients not included in this cohort might have being treated in other ways rather than by PCDT recommendation, such as by injunctions or administrative appeal.

## Conclusions

AMD patients are diagnosed late, after 70 years, most are white, female and received just 2.5 doses of antiangiogenic treatment within a year. There is still an important gap in AMD treatment in Brazil despite the standardization and incorporation of PCDT for AMD care, based on the few number of patients that received treatment according to its recommendation, few doses were done per patient, the lack of healthcare centers in some regions and the long distance traveled to reach AMD-care.

## Supplementary Information

**Additional file 1**: **Additional Table 1**. Distance evaluation from AMD patients’ residence to the healthcare institutions. **Additional Table 2**. Description of institutions that performed OCT among AMD patient since 2014.

## Data Availability

The raw datasets are available in the DATASUS repository, < http://www2.datasus.gov.br/DATASUS/index.php?area=0202 >. The processed and cleaned dataset are available from the corresponding author on reasonable request.

## Abbreviations

AMD: Age-related macular degeneration
BRL: Brazilian Real
CNES: Cadastro Nacional de Estabelecimentos de Saúde (National Register of Health Establishments)
nAMD: Neovascular age-related macular degeneration
OCT: Optical coherence tomography
PCDT: Protocolo Clínico e Diretrizes Terapêuticas
SAI: Sistema de Informações Ambulatoriais
SIH: Sistema de Informações Hospitalares
SUS: Sistema Único de Saúde
