# Supplementary material for: Age-related macular degeneration and resource utilization in the Brazilian public healthcare system: a real-world retrospective study
Source: BMC Ophthalmol. 2021 Dec 13;21:430. doi: 10.1186/s12886-021-02181-1 (PMC8667450; doi:10.1186/s12886-021-02181-1)
Supplement: Supplementary file 1 — Additional file 1: Additional Table 1. Distance evaluation from AMD patients’ residence to the healthcare institutions. Additional Table 2. Description of institutions that performed OCT among AMD patient since 2014. [file 12886_2021_2181_MOESM1_ESM.docx]

**Title:** Age-related macular degeneration and resource utilization in the Brazilian public healthcare system: a real-world retrospective study

**Authors**: Liane Touma Falci^1^, Carlos Augusto Moreira-Neto^2^, Alexandre Chater Taleb^3^, Marcela Bach Prieto^1^, Thais Packer^1^, Julio Cesar Barbour Oliveira^4^, Marina Gabriela Birck^4^, Guilherme Silva Julian^4^, Francisco Jose Forestiero^1^

Institutional addresses:

1. Novartis Biociências SA, São Paulo, SP, Brazil.

2. Hospital de Olhos do Paraná, Curitiba, PR, Brazil.

3. Reference Centre in Ophthalmology, Universidade Federal de Goiás, Goiânia, GO, Brazil.

4. IQVIA Brasil, São Paulo, SP, Brazil.

**Corresponding author**: Francisco Jose Forestiero

Address: Novartis Biociências SA Professor Vicente Rao Avenue, 90 São Paulo, SP, Brazil - 04636-000

Correspondence to [francisco.forestiero@novartis.com](mailto:francisco.forestiero@novartis.com)

**Additional files**

Additional table 1. Distance evaluation from AMD patients’ residence to the healthcare institutions.

|  | **Brazil** | **North** | **Northeast** | **Central-West** | **Southeast** | **South** |
| --- | --- | --- | --- | --- | --- | --- |
| **From residence to healthcare facility** | | | | | | |
| **Distance** (km), mean ± SD | 28.41 ± 75.60 | 14.66 ± 87.56 | 29.66 ± 154.76 | 37.66 ± 178.18 | 20.83 ± 59.19 | 53.76 ± 88.79 |
| **Distance,** N (%) |  |  |  |  |  |  |
| < 1 km | 54110 (2.5) | 1251 (2.0) | 14806 (23.6) | 5 (0.02) | 27590 (1.8) | 10458 (2.2) |
| 1 – 10 km | 1069568 (49.4) | 45019 (72.6) | 30550 (48.8) | 17072 (65.0) | 798205 (52.0) | 178722 (37.3) |
| 10 – 100 km | 921170 (42.5) | 15009 (24.2) | 13700 (21.9) | 8064 (30.7) | 665765 (43.3) | 218632 (45.6) |
| 100 – 1000 km | 119749 (5.5) | 512 (0.8) | 3235 (5.2) | 686 (2.6) | 43429 (2.8) | 71887 (15.0) |
| > 1000 km | 2185 (0.1) | 218 (0.4) | 354 (0.6) | 456 (1.7) | 1103 (0.1) | 54 (0.01) |
| **Missing information,** N (%) | 1118427 (34.0) | 12060 (16.2) | 47725 (43.2) | 26591 (50.2) | 529366 (25.6) | 502685 (51.1) |
| **From residence to tomography institution** | | | | | | |
| **Distance** (km), mean ± SD | 34.34 ± 67.66 | 20.57 ± 138.23 | 36.01 ± 121.0 | 20.69 ± 102.49 | 32.09 ± 54.48 | 59.79 ± 88.25 |
| **Distance,** N (%) |  |  |  |  |  |  |
| < 1 km | 353 (1.7) | 21 (3.9) | 112 (10.6) | 0 (0.0) | 184 (1.1) | 36 (1.9) |
| 1 – 10 km | 8198 (38.6) | 359 (67.5) | 534 (50.7) | 215 (65.2) | 6432 (37.0) | 658 (34.0) |
| 10 – 100 km | 11096 (52.3) | 143 (26.9) | 295 (28.0) | 109 (33.0) | 9582 (55.1) | 967 (50.0) |
| 100 – 1000 km | 1577 (7.4) | 6 (1.1) | 110 (10.4) | 4 (1.2) | 1185 (6.8) | 272 (14.1) |
| > 1000 km | 11 (0.1) | 3 (0.6) | 3 (0.3) | 2 (0.6) | 3 (0.0) | 0 (0.0) |
| **Missing information,** N (%) | 4920 (18.8) | 38 (6.6) | 679 (39.2) | 321 (49.3) | 2008 (10.3) | 1874 (49.2) |
| **From residence to antiangiogenic treatment institution** | | | | | | |
| **Distance** (km), mean ± SD | 64.85 ± 75.00 | - | - | - | 61.35 ± 62.84 | 59.79 ± 88.25 |
| **Distance,** N (%) |  |  |  |  |  |  |
| < 1 km | 10 (0.3) | - | - | - | 3 (0.1) | 7 (1.0) |
| 1 – 10 km | 681 (23.3) | - | - | - | 489 (22.3) | 192 (26.5) |
| 10 – 100 km | 1601 (54.8) | - | - | - | 1242 (56.6) | 359 (49.6) |
| 100 – 1000 km | 627 (21.5) | - | - | - | 461 (21.0) | 166 (22.9) |
| > 1000 km | 0 (0.0) | - | - | - | 0 (0.0) | 0 (0.0) |
| **Missing information,** N (%) | 453 (13.4) | - | - | - | 287 (83.4) | 57 (16.5) |

Additional table 2. Description of institutions that performed OCT among AMD patient since 2014, N (%).

|  | **Brazil** | **North** | **Northeast** | **Central-West** | **Southeast** | **South** |
| --- | --- | --- | --- | --- | --- | --- |
| **Number of institutions that performed tomography** | 94 | 5 (5.3) | 13 (13.8) | 4 (4.2) | 59 (62.8) | 13 (13.8) |
| **Type of institution** |  |  |  |  |  |  |
| General hospital | 36 (38.3) | 1 (20) | 2 (15.4) | 1 (25) | 24 (40.7) | 8 (61.5) |
| Specialized hospital | 11 (11.7) | 0 (0.0) | 4 (30.8) | 2 (50) | 5 (8.5) | 0 (0.0) |
| Clinics | 31 (33.0) | 3 (60) | 4 (30.8) | 1 (25) | 20 (33.9) | 3 (23.1) |
| Diagnostic Center | 2 (2.1) | 0 (0.0) | 0 (0.0) | 0 (0.0) | 2 (3.4) | 0 (0.0) |
| Day hospital | 14 (14.9) | 1 (20) | 3 (23.1) | 0 (0.0) | 8 (13.6) | 2 (15.4) |
| **Administrative type of hospital** |  |  |  |  |  |  |
| Regional hospital | 19 (52.8) | 0 (0.0) | 2 (100) | 1 (100) | 12 (50) | 4 (50) |
| State hospital | 13 (36.1) | 1 (100) | 0 (0.0) | 0 (0.0) | 12 (50) | 0 (0.0) |
| Double administration | 4 (11.1) | 0 (0.0) | 0 (0.0) | 0 (0.0) | 0 (0.0) | 4 (50) |
| **Size of institution** |  |  |  |  |  |  |
| Small (<50 beds) | 6 (16.7) | 0 (0.0) | 0 (0.0) | 1 (100) | 2 (8.3) | 3 (37.5) |
| Medium (51-150 beds) | 8 (22.2) | 1 (100) | 0 (0.0) | 0 (0.0) | 7 (29.2) | 0 (0.0) |
| Large (151-500 beds) | 15 (41.7) | 0 (0.0) | 1 (50) | 0 (0.0) | 11 (45.8) | 3 (37.5) |
| Extra-large (>500 beds) | 7 (19.4) | 0 (0.0) | 1 (50) | 0 (0.0) | 4 (16.7) | 2 (25) |
